# Prevalence and prognostic impact of abnormal left ventricular ejection fraction in Hemodialysis patients with end-stage renal disease

**DOI:** 10.1186/s12882-025-04316-8

**Published:** 2025-07-12

**Authors:** Qingkuan Li, Lingyue Qiu, Meiying Long, Huayuan Zeng, Zhihong Lu, Ling Liu, Yingzhong Lin, Kun Ye, Shaoming Qin, Qiuxia Wu, Qingwei Ji

**Affiliations:** 1 Jinan University, Guangzhou, 510632 China; 2 Guangxi Chest Pain Center, Department of Cardiology, Institute of Cardiovascular Diseases, The People’s Hospital of Guangxi Zhuang Autonomous Region, Guangxi Academy of Medical Sciences, Nanning, 530000 China; 3 Department of Nephrology, The People’s Hospital of Guangxi Zhuang Autonomous Region, Nanning, 530000 China

**Keywords:** End-stage renal disease, Left ventricular ejection fraction, Left ventricular systolic dysfunction, Hemodialysis

## Abstract

**Background:**

Patients with end-stage renal disease (ESRD) face a significantly elevated risk of cardiovascular morbidity and mortality, with left ventricular (LV) systolic dysfunction and heart failure (HF) being major contributors. Reduced left ventricular ejection fraction (LVEF) defines LV systolic dysfunction and is closely linked to adverse outcomes. This study aimed to assess the prevalence of abnormal LVEF in ESRD patients receiving hemodialysis and to examine the prognostic significance of varying LVEF levels on mortality and cardiovascular outcomes.

**Methods and results:**

A retrospective cohort study was conducted on 1,019 ESRD patients receiving hemodialysis at People’s Hospital of Guangxi Zhuang Autonomous Region between January 1, 2020, and December 31, 2021. Based on baseline LVEF, patients were classified into three groups: reduced ejection fraction (LVEF ≤ 40%, rEF), mildly reduced ejection fraction (LVEF 41–49%, mrEF), and normal ejection fraction (LVEF ≥ 50%, nEF). Clinical outcomes, including all-cause mortality and major adverse cardiovascular events (MACEs), were analyzed to assess the impact of LVEF levels. During a median follow-up of 35 months (IQR, 31–51 months), 214 patients (21.0%) died, and 218 (21.4%) experienced MACEs. The prevalence of abnormal LVEF was 13.35%, with 7.55% of patients in the mrEF group and 5.80% in the rEF group. Patients with abnormal LVEF showed significantly higher rates of all-cause mortality and MACEs than those with normal LVEF. In the rEF group, the odds ratios (ORs) for all-cause mortality and MACEs were 2.91 (95% CI: 1.83–4.63, *P* < 0.001) and 4.76 (95% CI: 2.43–9.46, *P* < 0.001), respectively. In the mrEF group, ORs for all-cause mortality and MACEs were 1.69 (95% CI: 1.09–2.62, *P* = 0.019) and 2.68 (95% CI: 1.54–4.68, *P* < 0.001), respectively.

**Conclusion:**

Abnormal LVEF is prevalent in ESRD patients on hemodialysis and is strongly associated with increased risks of all-cause mortality and MACEs. Lower LVEF levels correlate with poorer outcomes, underscoring the importance of early detection and targeted management strategies to improve prognosis in this high-risk population.

## Introduction

Chronic kidney disease (CKD) is a substantial global health burden and a major public health concern, affecting approximately 15% of adults worldwide in its most severe form, end-stage renal disease (ESRD) [1, 2]. As the primary treatment modality for ESRD, dialysis alleviates clinical symptoms and improves quality of life, yet dialysis-dependent patients continue to experience significantly elevated morbidity and mortality rates [3]. Cardiovascular disease (CVD) remains the leading cause of death in this population, with ESRD patients facing a 10- to 30-fold increased risk of cardiovascular mortality compared to the general population [4]. Nearly 50% of deaths among ESRD patients are attributed to CVD, with heart failure and sudden cardiac death being the most common causes [5]. In the United States, the prevalence of CVD in individuals over 65 years of age with CKD is 64.5%, significantly higher than the 32.4% prevalence in those without CKD [6, 7]. Given the high burden of cardiovascular complications, current clinical guidelines emphasize the importance of regular echocardiographic assessments for early detection and management of CVD in dialysis patients [8, 9].

Left ventricular (LV) systolic dysfunction is commonly observed in dialysis patients and is a well-established predictor of adverse outcomes [10]. According to the 2021 ESC guidelines for the diagnosis and treatment of acute and chronic heart failure [11] heart failure is categorized into three groups based on left ventricular ejection fraction (LVEF): normal LVEF (≥ 50%), mildly reduced LVEF (41–49%), and reduced LVEF (≤ 40%). Clinical outcomes vary significantly depending on LVEF in heart failure patients [12]. However, comprehensive studies investigating the association between varying LVEF levels and clinical outcomes in dialysis patients remain limited. This study retrospectively analyzes echocardiographic data from ESRD patients undergoing dialysis to evaluate the prevalence of abnormal LVEF and to investigate its association with all-cause mortality and major adverse cardiovascular events (MACEs). Findings from this study aim to inform the clinical management of dialysis patients with abnormal LVEF.

## Methods

### Study design and population

This retrospective cohort study included patients diagnosed with ESRD who were receiving dialysis at People’s Hospital of Guangxi Zhuang Autonomous Region between January 1, 2020, and December 31, 2021. ESRD was defined according to the Kidney Disease Outcomes Quality Initiative (KDOQI) guidelines as an estimated glomerular filtration rate (eGFR) of less than 15 mL/min/1.73 m² or initiation of dialysis therapy. Patients aged 18 years or older, diagnosed with ESRD and undergoing dialysis during the study period, were eligible for inclusion. Only patients with available echocardiographic evaluations from their first hospitalization during the study period were included. Exclusion criteria included a confirmed or suspected malignancy, a history of nephrectomy or kidney transplantation, significant missing clinical data (defined as missing key clinical information, such as left ventricular ejection fraction, medical history, and comorbidities, in more than 30% of patients), or loss to follow-up. The Ethics Committee of Guangxi Zhuang Autonomous Region People’s Hospital approved this study (KY-SY-2020-5), and all participants provided written informed consent in line with institutional guidelines. The study followed the Strengthening the Reporting of Observational Studies in Epidemiology (STROBE) reporting guidelines.

### Data collection

Detailed clinical data were collected, including demographic characteristics, medical history, and laboratory parameters. Dialysis-specific data encompassed modality (hemodialysis or peritoneal dialysis), duration, frequency, and medications used during dialysis. Echocardiographic data were acquired using a Vivid E9 color Doppler ultrasound system (GE Healthcare, USA). Parameters measured included left ventricular ejection fraction (LVEF), left ventricular end-diastolic diameter (LVEDD), left ventricular posterior wall thickness (LVPW), interventricular septal thickness (IVS), left ventricular mass indices (LVMI) and atrial dimensions (both left and right). The most recent echocardiographic data prior to patient enrollment were used for analysis.

### Endpoints

All patients were followed until May 8, 2024, for the assessment of primary and secondary endpoints. The primary endpoint was all-cause mortality. Secondary endpoints included MACEs, defined as a composite of cardiovascular death, non-fatal myocardial infarction, non-fatal stroke, and hospitalization due to heart failure. Cardiovascular death encompassed sudden cardiac death and deaths resulting from hypertensive disease, cerebrovascular disease, aortic disease, or complications related to cardiovascular conditions, such as acute myocardial infarction, atherosclerosis, and cardiomyopathy.

### Statistical analysis

Categorical variables were presented as counts and percentages, while continuous variables were expressed as means with standard deviations (SD) or medians with interquartile ranges (IQR), depending on distribution. Comparisons between categorical variables were conducted using the χ² test or Fisher’s exact test, while continuous variables were compared using the t-test or Kruskal-Wallis test, as appropriate. Kaplan-Meier survival curves were generated to estimate the cumulative survival probabilities across different LVEF categories. To assess the differences in survival between these categories, pairwise comparisons were performed using the log-rank test. The log-rank test was applied separately for each pairwise comparison. The Cox proportional hazards model was applied to assess the association between LVEF levels and mortality, while logistic regression was used to evaluate the impact of LVEF on the incidence of MACEs. Variables with significant associations in univariate analyses were included in multivariate models to estimate hazard ratios (HRs), odds ratios (ORs), and 95% confidence intervals (CIs) for LVEF categories concerning outcomes. Restricted cubic splines (RCS) were utilized to examine potential non-linear relationships between LVEF and HRs/ORs in dialysis patients. All statistical analyses were performed using R software version 4.1.0, with two-sided *P*-values < 0.05 indicating statistical significance.

## Results

### Prevalence and clinical risk factors of abnormal LVEF in dialysis patients

A total of 1,019 patients with ESRD were included in the analysis, with 646 (63%) being male. The median age of the cohort was 56 years (IQR, 43–65), and the median duration on dialysis was 1 month (IQR, 0–24). Hypertension was the most prevalent comorbidity, present in 80.5% of patients, followed by diabetes mellitus in 27.7%. The leading causes of ESRD in this cohort were hypertensive nephrosclerosis (31.2%) and diabetic nephropathy (26.9%), followed by obstructive nephropathy (12.1%) and chronic glomerulonephritis (9.8%). In 20.0% of patients, the underlying cause of ESRD remained unidentified due to insufficient diagnostic data available at the time of enrollment. Based on LVEF, patients were categorized into three groups: 883 patients (86.65%) had normal LVEF (≥ 50%), 77 patients (7.55%) had mildly reduced LVEF (41–49%), and 59 patients (5.80%) had reduced LVEF (≤ 40%). Patients with abnormal LVEF exhibited higher rates of smoking, alcohol consumption, and longer dialysis durations compared to those with normal LVEF. In addition, coronary artery disease, prior myocardial infarction, and history of coronary revascularization were more common among patients with abnormal LVEF. These patients also had elevated plasma levels of NT-proBNP and troponin. Clinically, a greater proportion of patients with abnormal LVEF were treated with angiotensin receptor-neprilysin inhibitors (ARNI) and β-blockers. Furthermore, patients with abnormal LVEF had significantly increased LVEDD and LVMI compared to those with normal LV function. Table 1 provides a detailed summary of baseline characteristics of dialysis patients stratified by LVEF levels.

**Table 1:** Baseline characteristics of dialysis patients by different LVEF values

Variable	LVEF ≥ 50%, (*n* = 883)	LVEF 41–49%, (*n* = 77)	LVEF ≤ 40% (*n* = 59)	*P*-value
Age (years)	55(15)	54 (16)	52 (16)	0.370
Gender (Male)	543 (61%)	53 (69%)	41 (69%)	0.231
BMI (kg/m²)	23.52 (4.09)	22.62 (4.00)	23.17 (4.12)	0.169
Smoking	86 (9.7%)	10 (13%)	12 (20%)	0.029
Alcohol Consumption	83 (9.4%)	12 (16%)	12 (20%)	0.009
Duration of Dialysis (months)	1 (0, 24)	4 (0, 24)	12(0.25, 36)	0.015
Comorbidities				
Coronary artery disease	60 (6.8%)	13 (17%)	12 (20%)	< 0.001
Myocardial infarction	20 (2.3%)	8 (10%)	6 (10%)	< 0.001
PCI/CABG	25 (2.8%)	8 (10%)	10 (17%)	< 0.001
Valvular heart disease	5 (0.6%)	2 (2.6%)	0 (0%)	0.147
Hypertension	717 (81%)	61 (79%)	42 (71%)	0.164
Diabetes mellitus	242 (27%)	24 (31%)	16 (27%)	0.775
Cerebrovascular disease	78 (8.8%)	5 (6.5%)	6 (10%)	0.723
COPD	4 (0.5%)	0 (0%)	1 (1.7%)	0.298
Medications during hospitalization				
ARNI (Sacubitril/Valsartan)	35 (4.0%)	8 (10%)	14 (24%)	< 0.001
ACEI/ARB	161 (18%)	16 (21%)	15 (25%)	0.354
Β-blockers	322 (36%)	36 (47%)	29 (49%)	0.039
SGLT2 inhibitors	4 (0.5%)	0 (0%)	2 (3.4%)	0.062
Calcium channel blockers	231 (26%)	20 (26%)	23 (39%)	0.097
Diuretics	177 (20%)	19 (25%)	19 (32%)	0.062
Statins	600 (68%)	50 (65%)	34 (58%)	0.240
HGB (g/L)	87.10 (23.64)	86.40 (24.35)	95.82 (21.32)	0.035
NT-proBNP (pg/mL)	6,452.00 (2,217.50, 22,232.00)	35,000.00 (8,599.25, 35,000.00)	35,000.00 (35,000.00, 35,000.00)	< 0.001
TNT_hs (ng/ml)	0.07 (0.04, 0.12)	0.12 (0.08, 0.21)	0.17 (0.10, 0.25)	< 0.001
LDL-c (mmol/L)	2.63 (2.13, 3.28)	2.48 (1.92, 3.33)	2.62 (2.22, 3.12)	0.49
Total Cholesterol (mmol/L)	3.61 (1.80)	3.62 (1.93)	3.44 (1.48)	0.938
Creatinine (µmol/L)	9.32 (6.73, 12.46)	8.05 (6.4, 11.44)	8.31 (5.49, 9.97)	0.027
Urea (mmol/L)	22.6 (17.4, 28.3)	21.49 (16.58, 27.48)	19.65 (14.59, 25.67)	0.052
Echocardiography				
EF (%)	63.18 (5.70)	45.43 (2.49)	33.19 (5.94)	< 0.001
LVEDD (mm)	49.94 (5.81)	56.77 (5.32)	62.05 (6.50)	< 0.001
IVS (mm)	11.31 (1.74)	11.38 (1.80)	11.12 (2.57)	0.616
LVPW (mm)	11.28 (1.75)	11.32 (1.83)	10.97 (2.36)	0.617
LVMI (g/m²)	203.10 (58.78)	271.63 (60.71)	315.78 (80.26)	< 0.001

Categorical variables: n (%), Continuous variables: Mean ± SD, Median (IQR) PCI: percutaneous coronary intervention; CABG: coronary artery bypass grafting; HGB: hemoglobin; LVEDD: left ventricular end-diastolic diameter; IVS: interventricular septum; LVPW: left ventricular posterior wall; LVMI: left ventricular mass index

### Differences in clinical outcomes among dialysis patients with varying LVEF levels

**Fig. 1:**
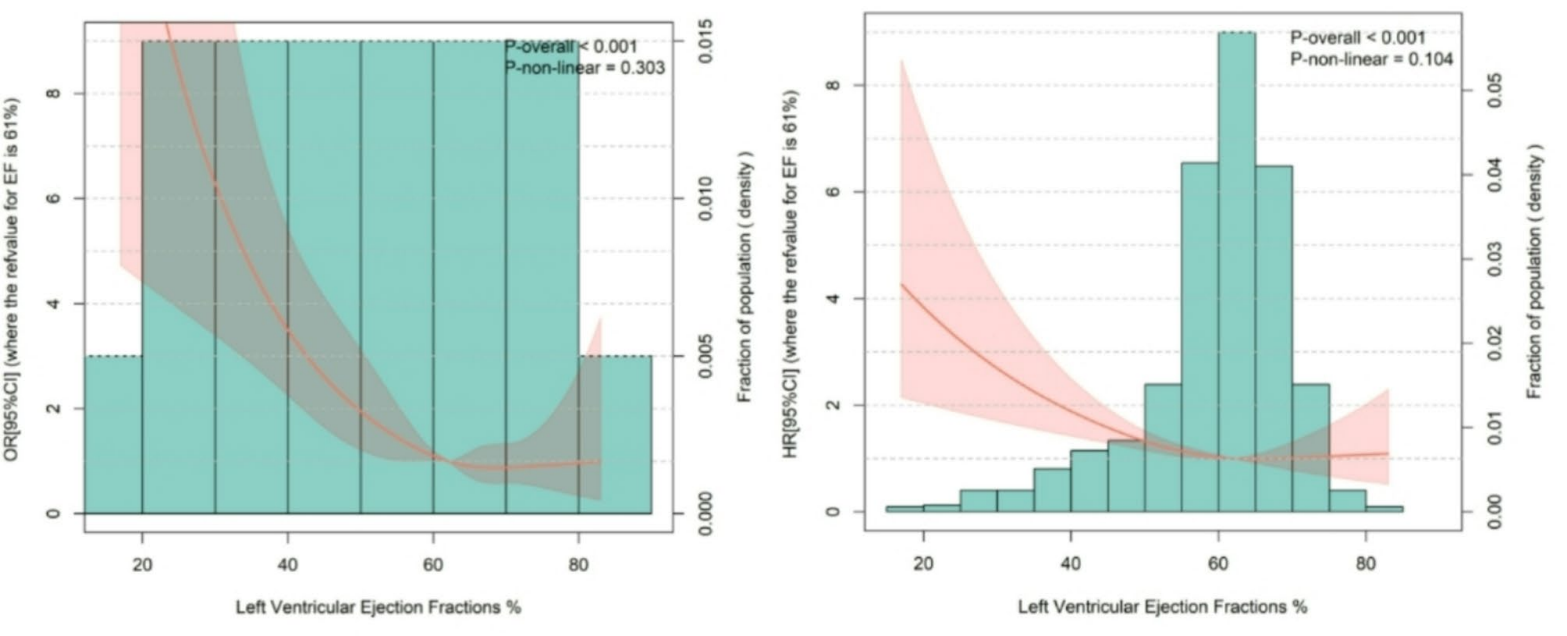
Relationship between LVEF and OR (**A**)/HR (**B**) in dialysis patients with ESRD. LVEF: Left Ventricular Ejection Fraction; ESRD: end stage renal disease; OR: odds ratio; HR: Hazard Ratio

**Fig. 2:**
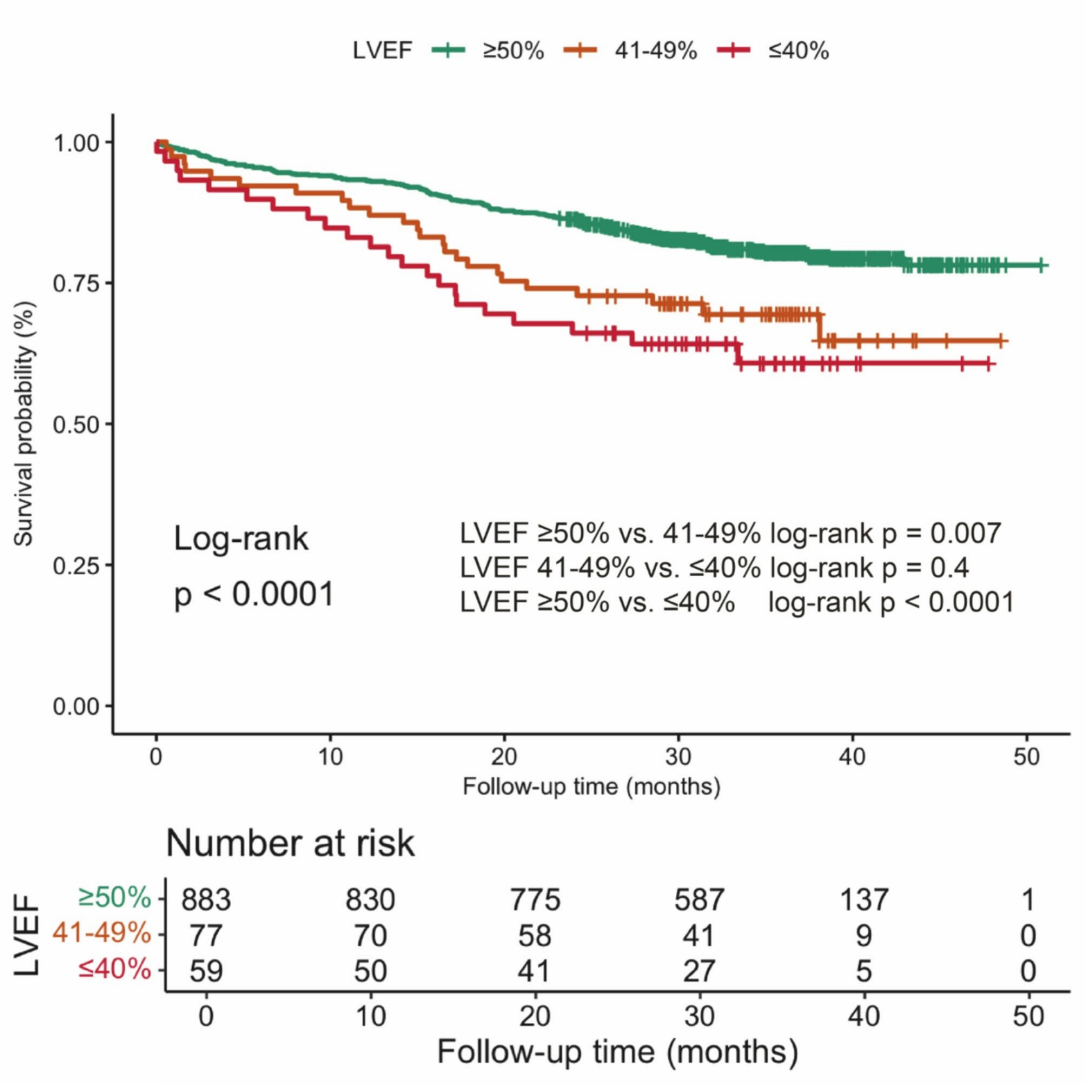
Survival curves comparison among dialysis patients with ESRD by different LVEF levels LVEF: Left Ventricular Ejection Fraction; ESRD: end stage renal disease

The median follow-up was 35 months (IQR, 31–51 months). During this period, 214 patients (21.0%) died, and 283 (28.0%) experienced MACEs, including 78 cases of cardiovascular death (7.7%), 27 non-fatal myocardial infarctions (2.6%), 41 non-fatal strokes (4.0%), and 196 hospitalizations for heart failure (19.0%), with heart failure hospitalization being the most frequently observed event. Both all-cause mortality and MACE rates showed a decreasing trend with increasing LVEF (Fig. 1). Patients with reduced LVEF experienced the highest incidence of both endpoints (Table 2; Fig. 2). The survival probability was significantly lower in patients with LVEF ≤ 40% compared to those with LVEF ≥ 50% (*P* < 0.0001). No significant difference was observed between the LVEF 41–49% and LVEF ≤ 40% groups (*P* = 0.4). Furthermore, patients with LVEF ≥ 50% demonstrated significantly better survival outcomes than those with LVEF 41–49% (*P* = 0.007) (Fig. 2). Significant differences were identified in both all-cause mortality and MACE incidence across the three LVEF groups. Patients with abnormal LVEF exhibited markedly higher rates of cardiovascular death, non-fatal myocardial infarction, and heart failure rehospitalization compared to those with normal LVEF (Table 3).

**Table 2:** Incidence of outcome events in dialysis patients

Endpoints, *n*(%)	Overall (*n* = 1019)	LVEF ≥ 50% (*n* = 883)	LVEF 41–49% (*n* = 77)	LVEF ≤ 40% (*n* = 59)	*P*-value
All-cause mortality	214 (21%)	168 (19%)	24 (31%)	22 (37%)	< 0.001
MACEs	283 (28%)	211 (24%)	36 (47%)	36 (61%)	< 0.001
Cardiovascular death	78 (7.7%)	54 (6.1%)	11 (14%)	13 (22%)	< 0.001
Non-fatal myocardial infarction	27 (2.6%)	19 (2.2%)	3 (3.9%)	5 (8.5%)	0.014
Non-fatal stroke	41 (4.0%)	37 (4.2%)	2 (2.6%)	2 (3.4%)	0.935
Rehospitalization for heart failure	196 (19%)	138 (16%)	28 (36%)	30 (51%)	< 0.001

MACEs: major adverse cardiovascular events, including non-fatal myocardial infarction, non-fatal stroke, and hospitalization due to heart failure

**Table 3:** Relationship between LV systolic dysfunction and prognosis in dialysis patients

Characteristics	Univariate Cox Analysis	*P*-value	Multivariate Cox Analysis	*P*-value
	HR (95CI%)		HR (95CI%)	
Demographic Characteristics				
Age (years)	1.06(1.05–1.07)	< 0.001	1.06(1.04–1.07)	< 0.001
Gender (Male vs. Female)	1.17(0.88–1.54)	0.287		
BMI	1.00(0.96–1.03)	0.820		
Smoking, n (%)	1.76(1.23–2.53)	0.002	1.61(1.12–2.32)	0.010
Alcohol Consumption, n (%)	1.10(0.72–1.69)	0.645		
Comorbidities, n (%)				
Coronary artery disease	2.87(2.02–4.07)	< 0.001	1.23(0.75–2.01)	0.400
Myocardial infarction	3.32(2.02–5.45)	< 0.001	2.00(0.94–4.26)	0.072
PCI/CABG	2.37(1.44–3.89)	0.001	0.47(0.22-1.00)	0.051
Valvular heart disease	2.75(0.88–8.59)	0.082		
Hypertension	1.25(0.87–1.79)	0.230		
Diabetes mellitus	2.23(1.7–2.92)	< 0.001	1.40(1.05–1.85)	0.020
Cerebrovascular disease	1.94(1.32–2.84)	0.001	1.10(0.74–1.63)	0.600
COPD	2.96(0.74–11.91)	0.127		
Echocardiography				
LVEF				
≥ 50%	reference		reference	
41–49%	1.79(1.17–2.75)	0.008	1.69(1.09–2.62)	0.019
≤ 40%	2.34(1.5–3.65)	< 0.001	2.91(1.83–4.63)	< 0.001
LVEDD	1.02(1-1.04)	0.123		
IVS	1.05(0.98–1.13)	0.172		
LVPW	1.03(0.96–1.11)	0.387		
LVMI	1.00(1.0–1.0)	0.999		

PCI: percutaneous coronary intervention; CABG: coronary artery bypass grafting; LVEDD: left ventricular end-diastolic diameter; IVS: interventricular septum; LVPW: left ventricular posterior wall; LVMI: left ventricular mass index

### Relationship between abnormal LVEF and prognosis in dialysis patients

Compared to patients with normal LVEF, those with mildly reduced or reduced LVEF had significantly higher mortality risks, with HRs of 1.79 and 2.34, respectively. In multivariate Cox regression analysis, adjusted for confounders such as age, smoking status, history of coronary artery disease, prior myocardial infarction, PCI or CABG history, diabetes, and cerebrovascular disease, patients with mildly reduced LVEF had a 1.69-fold increased risk of all-cause mortality compared to those with normal LVEF (95% CI: 1.09–2.62, *P* = 0.019). Patients with reduced LVEF had a 2.91-fold increased risk of all-cause mortality (95% CI: 1.83–4.63, *P* < 0.001) (Table 3). Furthermore, each decrement in LVEF was associated with a 74% increase in the risk of death (HR = 1.74, 95% CI: 1.40–2.17, *P-trend* < 0.001). Independent prognostic factors for all-cause mortality in dialysis patients included age, smoking, and diabetes (Table 4).

**Table 4:** Relationship between LV systolic dysfunction and cardiovascular adverse events in dialysis patients

Characteristics	Univariate Logistic Analysis	*P*-value	Multivariate Logistic Analysis	*P*-value
	OR (95CI%)		OR (95CI%)	
Demographic Characteristics				
Age (years)	1.04(1.03–1.05)	< 0.001	1.03(1.02–1.04)	< 0.001
Gender (Male vs. Female)	1.19(0.89–1.58)	0.243		
BMI	1.05(1.01–1.08)	0.006	1.04(1.00-1.08)	0.051
Smoking, n (%)	2.3(1.53–3.46)	< 0.001	1.92(1.05–3.53)	0.035
Alcohol Consumption, n (%)	1.8(1.19–2.72)	0.006	0.96(0.51–1.79)	0.999
Comorbidities, n (%)				
Coronary artery disease	3.86(2.45–6.07)	< 0.001	1.41(0.70–2.81)	0.300
Myocardial infarction	6.73(3.17–14.26)	< 0.001	2.47(0.87–7.31)	0.093
PCI/CABG	4.75(2.52–8.95)	< 0.001	0.93(0.35–2.45)	0.900
Valvular heart disease	2.83(1.82–4.39)	< 0.001	2.14(1.32–3.46)	0.002
Hypertension	1.31(0.91–1.87)	0.145		
Diabetes mellitus	2.28(1.7–3.06)	< 0.001	1.51(1.08–2.11)	0.016
Cerebrovascular disease	1.96(0.44–8.82)	0.380		
COPD	3.93(0.65–23.66)	0.135		
Echocardiography				
LVEF				
≥ 50%	reference		reference	
41–49%	2.8(1.74–4.49)	< 0.001	2.68(1.54–4.68)	< 0.001
≤ 40%	4.98(2.89–8.6)	< 0.001	4.76(2.43–9.46)	< 0.001
LVEDD	1.05(1.03–1.07)	< 0.001	1.01(0.96–1.05)	0.800
IVS	1(0.92–1.08)	0.957		
LVPW	0.99(0.92–1.07)	0.819		
LVMI	1(1-1.01)	< 0.001	1(1.00-1.01)	0.500

PCI: percutaneous coronary intervention; CABG: coronary artery bypass grafting; LVEDD: left ventricular end-diastolic diameter; IVS: interventricular septum; LVPW: left ventricular posterior wall; LVMI: left ventricular mass index

### Relationship between abnormal LVEF and adverse cardiovascular events in dialysis patients

Univariate logistic analysis demonstrated that patients with mildly reduced or reduced LVEF had significantly higher risks of MACEs compared to those with normal LVEF (*P* < 0.001). In multivariate analysis, adjusted for confounders including age, sex, smoking, alcohol consumption, history of coronary artery disease, myocardial infarction, PCI or CABG, diabetes, cerebrovascular disease, and LVEDD, patients with mildly reduced LVEF had a 168% increased risk of MACE (OR = 2.68, 95% CI: 1.54–4.68, *P* < 0.001), while those with reduced LVEF had a 376% increased risk of MACE (OR = 4.76, 95% CI: 2.43–9.46, *P* < 0.001) (Table 4)). Trend analysis in the multivariate model showed that each stepwise decrease in LVEF was associated with a 128% increased risk of MACE (HR = 2.28, 95% CI: 1.66–3.14, *P-trend* < 0.001). Additionally, older age, smoking, and a history of cerebrovascular disease were significant predictors of MACE (*P* < 0.05), while body mass index (BMI) showed a trend toward significance (*P* = 0.051), suggesting a potential but not definitive association (Table 4).

## Discussion

In this single-center, retrospective observational study, we identified a relatively high prevalence of abnormal LVEF among patients undergoing dialysis for ESRD. Approximately 13.55% of these patients, who had a median dialysis duration of just 1 month, exhibited abnormal LVEF. This group faced significantly elevated risks of all-cause mortality and MACEs. Over a median follow-up of 35 months, the all-cause mortality rate reached 21.0%, with cardiovascular deaths accounting for 36.4% of all deaths. The incidence of MACEs was 28.0%, with heart failure-related rehospitalization being the most frequent event, occurring in 19.0% of patients. Importantly, abnormal LVEF was identified as an independent predictor of both all-cause mortality and MACEs. The lower the LVEF, the higher the risk of mortality and adverse cardiovascular outcomes, underscoring the significant prognostic value of abnormal LVEF in this population.

The elevated prevalence of CVD in dialysis patients can be attributed to an accumulation of traditional and dialysis-related cardiovascular risk factors [13, 14]. In addition to the pre-existing burden of hypertension and diabetes commonly found in ESRD, the dialysis process itself introduces hemodynamic stressors that may accelerate cardiovascular deterioration [15]. The rapid fluctuations in blood flow and electrolyte balance during dialysis, both during the procedure and before and after treatment, are considered key contributors to cardiovascular complications. These abrupt changes can lead to drops in blood pressure and disturbances in electrolyte levels, which in turn cause substantial myocardial damage, potentially resulting in severe outcomes such as cardiac arrest and heart failure. Our findings align with previous studies by Yamada et al. [3] and the CRIC study [16, 17]both of which highlighted the high prevalence of abnormal LVEF and its associated risks in patients with advanced CKD and those newly initiated on dialysis. In our cohort, 7.55% of patients had mildly reduced LVEF (41–49%) and 5.80% had reduced LVEF (≤ 40%), consistent with Yamada’s study, which reported 13% of newly dialyzed patients with LVEF below 50%. Patients with abnormal LVEF in our study had longer dialysis durations, a higher prevalence of coronary artery disease, and elevated biomarkers (NT-proBNP and troponin) compared to those with normal LV function, reinforcing the association between abnormal LVEF and cardiovascular comorbidities in dialysis patients. The coexistence of traditional cardiovascular risk factors, such as hypertension and diabetes, compounded by chronic volume overload, anemia, arteriovenous shunting, and uremic toxicity, significantly contributes to the structural and functional cardiac abnormalities observed in ESRD patients [18]. The identification of the underlying etiology of ESRD is clinically important, as it may influence both cardiovascular risk profiles and long-term outcomes. In our cohort, hypertensive nephrosclerosis and diabetic nephropathy were the most prevalent causes of ESRD—both of which are known to be strongly associated with elevated cardiovascular morbidity and mortality. The predominance of these high-risk etiologies may partially account for the substantial burden of cardiovascular events observed in our population.

The impact of LVEF on mortality and adverse cardiovascular outcomes in dialysis patients aligns with prior studies, which have shown increased mortality in the presence of heart failure or LV systolic dysfunction [19–21]. For example, dialysis patients with heart failure have been shown to have a markedly lower 2-year survival rate compared to those without heart failure (33% vs. 80%) [20]. Similarly, patients with LVEF > 45% were shown to have significantly better survival compared to those with LVEF ≤ 45% (73% vs. 55%) [22]. In our study, the all-cause mortality rate over a median follow-up of 35 months was 21%, with cardiovascular deaths accounting for 36.4% of all deaths. Patients with LVEF < 50% had an all-cause mortality rate of 33.8%, substantially higher than the 19% observed in patients with normal LVEF, aligning with previous research. Our study’s findings further detail this relationship by categorizing patients with abnormal LVEF into mildly reduced (41–49%) and reduced (≤ 40%) subgroups, revealing a stepwise increase in mortality with decreasing LVEF. Specifically, the all-cause mortality rates were 31% in the mildly reduced group and 37% in the reduced group, suggesting that each incremental decline in LVEF is associated with progressively poorer outcomes. The MACE incidence also escalated with worsening LVEF: from 24% in patients with normal LVEF (≥ 50%) to 47% and 61% in those with mildly reduced and reduced LVEF, respectively. Multivariate analysis further confirmed that abnormal LVEF was an independent predictor of both all-cause mortality and MACEs, emphasizing the importance of precise LVEF stratification in risk assessment for dialysis patients.

The interplay between ESRD and LV systolic dysfunction is well-documented [23]. LV systolic dysfunction can lead to reduced renal perfusion, stimulating the renin-angiotensin system and exacerbating sodium and water retention, ultimately contributing to volume overload and impaired renal function. This cycle perpetuates a worsening state of both heart and kidney function [24]. Conversely, ESRD itself imposes chronic hemodynamic stress due to volume overload, hypertension, anemia, and the toxic effects of uremia, which together aggravate cardiac dysfunction. The elevated mortality and MACE rates observed in our study, particularly among patients with abnormal LVEF, likely reflect these combined pathophysiological stresses. These findings highlight the need for regular cardiovascular evaluation, especially echocardiography, in dialysis patients. Early detection and targeted management of abnormal LVEF may help mitigate the adverse outcomes associated with these complex interactions. Our study primarily focused on Chinese patients with ESRD, and it is essential to recognize that race, genetic polymorphisms, and hereditary factors significantly influence cardiovascular risk in this population. Moreover, the dialysis population is inherently heterogeneous, with significant variations in key factors such as age, comorbidities, dialysis duration, blood pressure control, and other cardiovascular risk factors. These variations must be considered when translating the findings to other populations. Future research should adopt multicenter, multi-ethnic study designs to investigate the similarities and differences across diverse populations, thereby enhancing our understanding of cardiovascular risks in ESRD patients and facilitating the development of more personalized treatment strategies.

### Limitations

Several limitations should be acknowledged. First, the retrospective and single-center design may limit the generalizability of our findings. Larger, multicenter studies would help validate and expand upon these results. Second, our study lacked comprehensive data on volume-related parameters such as dry weight and ultrafiltration, which may impact patient outcomes. Consequently, we were unable to assess the effect of volume control on echocardiographic measures. Although we demonstrated the prognostic impact of abnormal LVEF, we did not assess the role of heart failure therapies in this specific population. Future studies should evaluate the efficacy of guideline-recommended heart failure therapies in dialysis patients with abnormal LVEF to identify potential therapeutic strategies.

Additionally, although we demonstrated the prognostic impact of abnormal LVEF, we did not assess the role of heart failure therapies in this specific population. Future studies should evaluate the efficacy of guideline-recommended heart failure therapies in dialysis patients with abnormal LVEF to identify potential therapeutic strategies.

## Conclusion

Our study demonstrates that abnormal LVEF is a common complication among dialysis patients, significantly associated with increased all-cause mortality and MACEs. Cardiovascular deaths accounted for a significant proportion of mortality, with heart failure being the most frequent cardiovascular event. Multivariate analysis confirmed that abnormal LVEF is an independent predictor of both mortality and MACEs. These findings highlight the importance of early detection and management of abnormal LVEF to improve the prognosis of dialysis patients.

## Data Availability

The datasets used during the current study are available from the corresponding author on reasonable request.
